# Epigenomics coverage data extraction and aggregation in R with tidyCoverage

**DOI:** 10.1093/bioinformatics/btae487

**Published:** 2024-07-29

**Authors:** Jacques Serizay, Romain Koszul

**Affiliations:** Institut Pasteur, CNRS UMR 3525, Université Paris Cité, Unité Régulation Spatiale des Génomes, Paris 75015, France; Institut Pasteur, CNRS UMR 3525, Université Paris Cité, Unité Régulation Spatiale des Génomes, Paris 75015, France

## Abstract

**Summary:**

The tidyCoverage R package provides a framework for intuitive investigation of collections of genomic tracks over genomic features, relying on the principle of tidy data manipulation. It defines two data structures, CoverageExperiment and AggregatedCoverage classes, directly extending the SummarizedExperiment fundamental class, and introduces a principled approach to exploring genome-wide data. This infrastructure facilitates the extraction and manipulation of genomic coverage track data across individual or multiple sets of thousands of genomic loci. This allows the end user to rapidly visualize track coverage at individual genomic loci or aggregated coverage profiles over sets of genomic loci. tidyCoverage seamlessly combines with the existing Bioconductor ecosystem to accelerate the integration of genome-wide track data in epigenomic analysis workflows. tidyCoverage emerges as a valuable tool, contributing to the advancement of epigenomics research by promoting consistency, reproducibility, and accessibility in data analysis.

**Availability and implementation:**

tidyCoverage is an R package freely available from Bioconductor ≥ 3.19 (https://www.bioconductor.org/packages/tidyCoverage) for R ≥ 4.4. The software is distributed under the MIT License and is accompanied by example files and data.

## 1 Introduction

Genome-wide epigenomic assays provide powerful methods to profile chromatin composition, conformation and activity. Linear “coverage” tracks are one of the main output files obtained when processing sequencing data. These coverage tracks, generally stored as *.*bigwig files, are often inspected in genome interactive browsers (e.g. IGV) to visually appreciate local or genome-wide variations in the coverage of specific genomic assays. Another approach to investigate genomic tracks is to compute and plot the average profile of a genomic track over a set of genomic loci. This approach is very efficient to summarize and compare the coverage of chromatin modalities (e.g. protein binding profiles from ChIP-seq, transcription profiles from RNA-seq, chromatin accessibility from ATAC-seq, etc.) over hundreds and up to thousands of genomic features of interest. This can be used to accurately describe, both qualitatively and quantitatively, multi-omic genomic tracks summarized across multiple sets of genomic features.

To create such metaplots, a number of tools already exist in a command-line interface—e.g. deeptools (Ramírez *et al.* 2016)—or as packages in R—e.g. genomation (Akalin *et al.* 2015), ATACseqQC (Ou *et al.* 2018) or soGGI (Dharmalingam n.d.). However, these tools (i) are not interconnected to existing bioinformatic resources, (ii) do not efficiently leverage the Bioconductor ecosystem and (iii) do not use a tidy, intuitive syntax for data processing (Wickham *et al.* 2019, Hutchison *et al.* 2024). Here, we present tidyCoverage, an R package extending Bioconductor fundamental data structures and reusing principles of tidy data manipulation to extract and aggregate coverage tracks over multiple sets of genomic features.

## 2 Implementation

### 2.1 Two new S4 classes implemented from *SummarizedExperiment*

**Figure 1.**
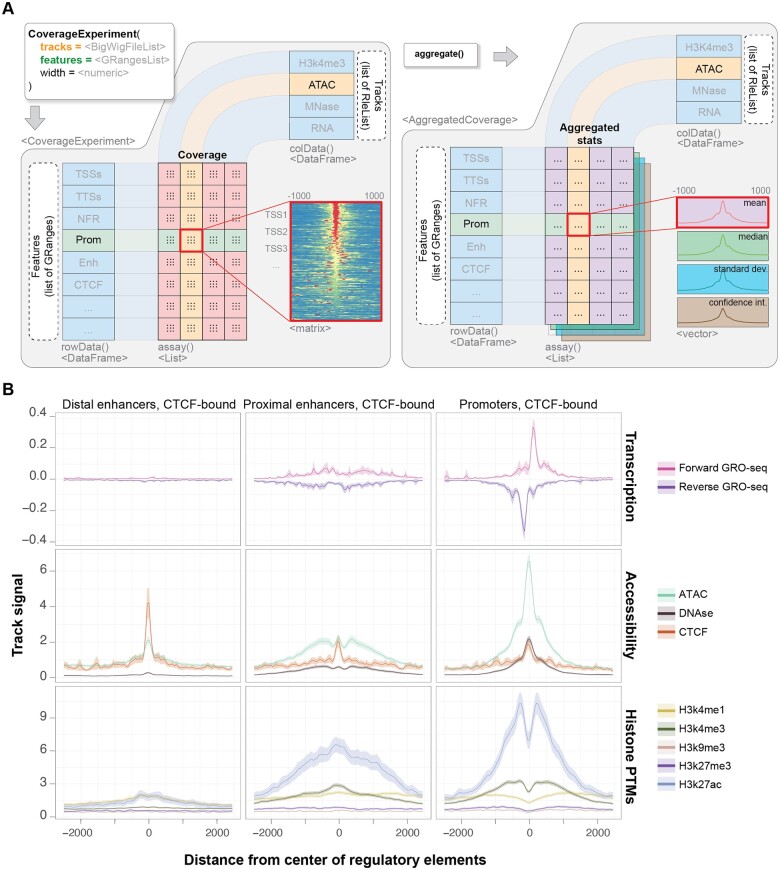
Overview of tidyCoverage functionalities. (A) The CoverageExperiment object extracts and stores a separate coverage matrix for pairs of genomic track and genomic features. It can be further aggregated into a AggregatedCoverage object, which stores statistical metrics (mean, min, max, median, standard deviation, confidence interval) of the coverage of each track over each set of genomic features. (B) tidyCoverage can be leveraged in combination with ggplot2 functionalities to produce advanced aggregated coverage plots, for multiple tracks and genomic features.

tidyCoverage implements the CoverageExperiment and AggregatedCoverage classes, both of which are built on top of the SummarizedExperiment class (Fig. 1A). This ensures seamless creation and manipulation of these objects by end users, in particular those already familiar with popular packages built on top of SummarizedExperiment, such as DESeq2 (Love *et al.* 2014) and SingleCellExperiment* (*Amezquita *et al.* 2020). CoverageExperiment objects organize a collection of genome-wide tracks (from local *.*bigwig files or numerical tracks stored in memory) and a collection of sets of genomic features of interest. When instantiated, the coverage of each genomic track is extracted using advanced Bioconductor parallelization and import infrastructures (Lawrence *et al.* 2009) and stored in memory as a numerical array. Importantly, coverage data are specifically extracted over the genomic features rather than across entire genomes. This allows fast and memory-efficient parsing of genomic files. AggregatedCoverage further computes statistical metrics (e.g. mean, median, standard deviation, etc.) from the coverage vectors stored in a CoverageExperiment object. tidyCoverage data structures are natively compatible with other genomic data representations (e.g. GenomicRanges, RleList, OrgDb) and facilitate the integration of epigenomic data into large-scale multi-omics projects.

### 2.2 Tidy principles for epigenomics

Tidy analysis of omics data has recently gained traction in large communities of bioinformaticians and programming languages (Hutchison *et al.* 2024), and tidyCoverage fully adheres to the tidy data paradigm. The package supports operative verbs defined in the tidyverse, such as filter, mutate, group_by or expand for CoverageExperiment and AggregatedCoverage objects. This enables researchers to efficiently organize, manipulate, and visualize epigenomic datasets in a tidy and structured format. tidyCoverage streamlines the intuitive exploration of large epigenomics datasets and facilitates data visualization using robust tools such as ggplot2.

## 3 Case study

To demonstrate the usability of tidyCoverage package, we recovered 10 different epigenomic profiles in the human cell line GM12878 from the ENCODE data portal (Luo *et al.* 2020): (i) GRO-seq (global run-on sequencing) stranded coverage tracks show forward and reverse nascent transcription, (ii) ATAC-seq, DNAse-seq and CTCF ChIP-seq yield information regarding the local chromatin accessibility, and (iii) ChIP-seq for post-translational modifications of different H3 lysine residues indicates chromatin composition. We used tidyCoverage to extract track coverage over tens of thousands of ENCODE-annotated cis-regulatory elements (ENCODE Project Consortium *et al.* 2020), grouped as promoters, proximal enhancers or distal enhancers (located >2kb from promoters), bound by CTCF. Aggregating epigenomic coverage tracks highlighted the different composition, structure and activity of the chromatin which makes up different types of regulatory elements (Fig. 1B). For instance, this reveals that CTCF enrichment is greater at distal enhancers than at proximal enhancers or promoters. This raises hypotheses regarding the implication of CTCF for chromatin looping and spatial folding at these different classes of regulatory elements.

## 4 Discussion

Compared to existing solutions, tidyCoverage focuses on data recovery and manipulation, using a standard representation of the data and principles of tidy data manipulation. tidyCoverage also ensures seamless integration of genomic track data into the existing genomics-centric Bioconductor ecosystem. This will contribute to the advancement of epigenomics research by fostering efficient and reproducible analyses.

## Supplementary Material

btae487_Supplementary_Data

## Data Availability

All data presented in this manuscript have already been published. Human ENCODE-annotated regulatory elements were retrieved from (ENCODE Project Consortium *et al.* 2020) (Supplementary Table S10). The genomic tracks were retrieved from the ENCODE data portal from the following IDs: forward GRO-seq: ENCFF896TNM; reverse GRO-seq: ENCFF764SVR; Pol2RA ChIP-seq: ENCFF890SYC; CTCF ChIP-seq: ENCFF484SOD; DNAse-seq: ENCFF428XFI; ATAC-seq: ENCFF165WGA; H3K4me1 ChIP-seq: ENCFF785YET; H3K4me3 ChIP-seq: ENCFF736DCK; H3K9me3 ChIP-seq: ENCFF698SKV; H3K27me3 ChIP-seq: ENCFF119CAV; H3K27ac ChIP-seq: ENCFF458CR.

## Code availability

The entire code used to generate Fig. 1B is available here: https://github.com/js2264/tidyCoverage/blob/cc28588/vignettes/manuscript_figure.qmd.
